# Two distinct types of the inhibition of vasculogenesis by different species of charged particles

**DOI:** 10.1186/2045-824X-5-16

**Published:** 2013-09-17

**Authors:** Peter Grabham, Preety Sharma, Alan Bigelow, Charles Geard

**Affiliations:** 1 Center for Radiological Research, Columbia University, VC 11-205A/243, 630 West 168th street, New York, NY 10032, USA; 2 Radiological Research Accelerator Facility, Center for Radiological Research, Nevis Laboratory, Columbia University, 136 S. Broadway, Irvington, NY 10533, USA

**Keywords:** Vasculogenesis, Charged particle, Radiation, Linear energy transfer, 3-D human vessel models, Motile tips

## Abstract

**Background:**

Charged particle radiation is known to be more biologically effective than photon radiation. One example of this is the inhibition of the formation of human blood vessels. This effect is an important factor influencing human health and is relevant to space travel as well as to cancer radiotherapy. We have previously shown that ion particles with a high energy deposition, or linear energy transfer (LET) are more than four times more effective at disrupting mature vessel tissue models than particles with a lower LET. For vasculogenesis however, the relative biological effectiveness between particles is the same. This unexpected result prompted us to investigate whether the inhibition of vasculogenesis was occurring by distinct mechanisms.

**Methods:**

Using 3-Dimensional human vessel models, we developed assays that determine at what stage angiogenesis is inhibited. Vessel morphology, the presence of motile tip structures, and changes in the matrix architecture were assessed. To confirm that the mechanisms are distinct, stimulation of Protein Kinase C (PKC) with phorbol ester (PMA) was employed to selectively restore vessel formation in cultures where early motile tip activity was inhibited.

**Results:**

Endothelial cells in 3-D culture exposed to low LET protons failed to make connections with other cells but eventually developed a central lumen. Conversely, cells exposed to high LET Fe charged particles extended cellular processes and made connections to other cells but did not develop a central lumen. The microtubule and actin cytoskeletons indicated that motility at the extending tips of endothelial cells is inhibited by low LET but not high LET particles. Actin-rich protrusive structures that contain bundled microtubules showed a 65% decrease when exposed to low LET particles but not high LET particles, with commensurate changes in the matrix architecture. Stimulation of PKC with PMA restored tip motility and capillary formation in low but not high LET particle treated cultures.

**Conclusion:**

Low LET charged particles inhibit the early stages of vasculogenesis when tip cells have motile protrusive structures and are creating pioneer guidance tunnels through the matrix. High LET charged particles do not affect the early stages of vasculogenesis but they do affect the later stages when the endothelial cells migrate to form tubes.

## Background

The importance of our understanding of the effects of powerful ion particle radiation on humans becomes greater as more people spend more time in the space environment, and as carbon ion (C^+^) and proton (H^**+**^) radiotherapy become increasingly used in the treatment of cancer. Ion particles are more biologically effective than photons such as gamma rays, and unlike photons, they penetrate tissue in a track structure oriented from the source. The effectiveness of these particles depends on their energy and mass, which determines the extent of energy deposition per unit of track length or the linear energy transfer (LET). As the nuclear particles penetrate matter they collide with other particles, produce secondary irradiations, and deposit energy in a penumbra around the track. The total energy deposited per mass is the absorbed dose (measured in Gray). A feature of particle radiation is that as the particle traverses matter it remains at a constant speed and energy deposition until it reaches a point where it slows down. Correspondingly, the LET increases to higher values until the particle eventually stops. Plotted over distance the energy deposition produces a Bragg curve where the LET remains at a plateau until the LET increases with the highest value at a peak near the end of the track [1]. Most studies however, (including the present study) irradiate samples when the particles are in the plateau stage of the Bragg curve and have a constant LET as they pass through the sample.

High-energy protons have a low LET similar to that of photon radiations whereas high-Z and energy (HZE) nuclei such as iron ions (Fe^**+**^) have a high LET and therefore a much greater ionization potential, with important qualitative and quantitative differences in their biological effects. High LET particles are generally more biologically effective than low LET particles as exemplified by DNA damage leading to cancer risk [2]. The biological effectiveness is expressed in terms of relative effect to a standard radiation like gamma rays giving the relative biological effectiveness (RBE).

Both high and low LET radiations exist in the space environment and in ion particle cancer radiotherapy. Space radiation is comprised of a complex mix of ionized atomic nuclei from helium to iron [3,4]. Although HZE particles are much less abundant than protons, their greater LET, and therefore relative biological effectiveness, makes them major contributors to the total dose equivalent (average measure of absorbed dose in a mass of tissue with weighting for different types of radiation) with Fe ions being the principal contributor [5]. Solar particle events consisting of relatively large doses of mixed LET protons also contribute to the radiation encountered in space. For radiotherapy, low LET ion particles are utilized at proton facilities, and higher LET charged particles are utilized by Carbon ion facilities.

The vast network of micro-capillaries and other vessels in human tissues make it a major target for the effects of radiation. Among these risks are diseases of the vasculature such as heart disease and stroke. Atomic bomb survivors show non-cancer disease mortality, including vascular diseases [6], and astronauts present with several physiological changes, including cardiovascular degeneration and adaptation [7]. In addition, cancer radiotherapy can cause peripheral artery disease, and the associated morphological changes are identical to those found in spontaneous atherosclerosis [8]. Experimental studies on the effects of ion particles on the vasculature are relatively few although they all indicate that vessel formation is inhibited by ion particles. [9-13]. In our investigations using human 3-Dimensional vessel models we found that low LET protons inhibited vessel formation (vasculogenesis in this model) with an equal biological effectiveness compared to high LET Fe ions. This result was unexpected considering the fact that Fe ions were at least four times more effective at disrupting mature vessels from the same model. In the present study we investigated whether this could be explained by low and high LET ions inhibiting vasculogenesis by distinct mechanisms.

## Methods

### Reagents and cell culture

Primary Human Brain Microvascular Endothelial Cells (HBMEC) were obtained from Cell Systems (Kirkland, WA). Human umbilical vein cells (HUVEC) Endothelial cell media, EBM and EBM-2 were obtained from Lonza Walkersville, Inc (Walkersville, MD). EBM medium for growth of undifferentiated HBMEC’s contains EGM medium (serum free, growth-factor free), supplemented with 2% fetal bovine serum (FBS), human epidermal growth factor, hydrocortisone, bovine brain extract. EBM-2 medium for vessel formation contains EGM-2 medium (serum free, growth-factor free), supplemented with 2% fetal bovine serum (FBS), human fibroblast growth factor-B (hFGF-B), human epidermal growth factor (hEGF), human vascular endothelial cell growth factor (hVEGF), long R insulin-like growth factor-1 (R3-IGF-1), ascorbic acid, hydrocortisone, and heparin.

In this study, 3D cell culture was performed according to the method initially described by Davis and Camarillo [14] with modifications described in Grabham et al., [11]. Briefly, Collagen gel solution was prepared on ice by mixing together the following stock solutions: 0.35% collagen solution, 10X M199 medium, and 1 M HEPES (pH 7.4) in a ratio of 8:1:1 by (volume). This solution was then mixed with matrigel (BD Biosciences, Bedford, MA) in a ratio of 3:1 by (volume). HBMEC grown in EGM on 2D cell culture dishes (80-90% confluence) were detached with a trypsin solution (0.025%). and resuspended in EGM2. The cell solution was mixed with the gel solution in a ratio of 1:5 (by volume). The resulting cell suspension contained a final cell density of 1 × 10^6^ cells/ml. 25 microliters was dropped onto the cell growing surface inside a tissue culture flask (T25) and allowed to gel at 37°C for 30 min. The gel matrices were then overlaid with EGM-2 medium containing 50 nM Phorbol 12-Myristate 13-acetate (PMA) (Sigma, St Louis, MO). Cells embedded in gel matrices with the dimensions of 4 mm diameter and 2 mm depth were incubated at 37°C in a humidified incubator (5% CO2, 95% air) for the times indicated. The medium was refreshed partially every 24 h or completely every 48 hours. Mature vessel models were developed after 6 days.

### Irradiation of vessel models

Iron-ion (1 GeV/nucleon; LET 151 keV/μm) or proton (1GeV/nucleon; LET 0.22 keV/μm irradiation was conducted at the NASA Space Radiation Laboratory (NSRL) at Brookhaven National Laboratory (BNL, Upton, NY) at a dose of 1Gy and at dose rates of 1 Gy/min for 1minute. Samples were placed in the plateau region of the Bragg curve and irradiated at room temperature. Dosimetry was performed by the NSRL physics staff. Since the heavy-ion beam at NSRL is horizontal, flasks containing 5 ml of medium were upended to a vertical position for a few minutes during irradiation.

### Immunocytochemistry and imaging

Vessel cultures were fixed at the appropriate times by the addition of 5 ml of PBS, pH 7.4, containing 4% paraformaldehyde, for 5 min at 37°C, followed by one rinse and three 5 min washes in PBS and 0.5% Triton X-100. For morphology studies, vessel structure was visualized by staining for all proteins using 5-(4,6-Dichlorotriazinyl) Aminofluorescein (5-DTAF) and nuclei were visualized with propidium iodide (Invitrogen, Carlsbad, CA). At least 10 fields in at least 2 separate experiments were assessed. The actin cytoskeleton was visualized using Alexa Fluor Phalloidin at a concentration of 1:40 in PBS (Invitrogen, Carlsbad, CA). Microtubules were immunostained using a monoclonal antibody to β tubulin (TBN06) at a dilution of 1:50 in PBS (Neomarkers, Freemont, CA) followed by Alexa fluor conjugates 495 or 488 at a dilution of 1:1000 (Invitrogen, Carlsbad, CA). Images were captured on a Nikon TE 200 confocal C1 microscope with EZ-C1 software. 10 Z planes 2 μm apart were captured for each field. Analysis of Z-projected images was carried out using NIH image software and a threshold, outline algorithm. Vessel development was determined by measuring the total length of vessel with lumens in each field divided by the number of cells in order to express these values as the total length per cell. Motile tips were determined by the following criteria; a cellular projection that is at least 30 μm long and contains bundled microtubules with motile actin structures – filopodia closely associated with the microtubules. Double staining shows overlap between actin and microtubules. For verification of microtubule bundling when needed plot profiles were carried out using the NIH image plot profile function. A single peak of pixel intensity (grey value) denotes bundled microtubules.

Two-photon and second harmonic generation (SHG) microscopy provided additional images for visualization of the collagen gel matrix. The excitation light source is a Chameleon Ultra II (Coherent Inc., Santa Clara, CA) tunable Titanium Sapphire laser whose infrared wavelengths have greater penetration depth and reduced photobleaching when compared with the excitation wavelengths required for traditional confocal microscopy. Signal from two-photon excitation or SHG was gathered through a 60X water-immersion objective and filtered by a dichroic mirror and appropriate emission filters placed in front of photo-multiplier tubes. 3D image information was obtained by acquiring a z-stack of optical sections with a z-step of 0.3 μm. The SHG imaging mode of our multiphoton microscope correlates the scanning position of the tunable excitation laser (780 nm wavelength) with detection of SHG signal (390 nm wavelength) to form an image. Various projections of the 3D images were accessed through blind deconvolution routines in NIH Image and in AutoQuant deblurring software (Media Cybernetics, Bethesda, MD).

## Results

### Distinct vessel morphologies after exposure to charged particles

**Figure 1:**
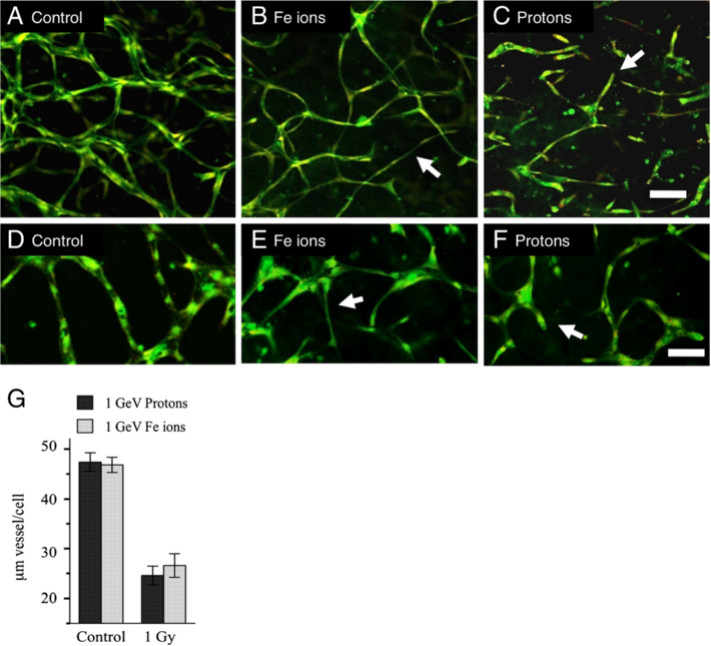
**Exposure to protons and Fe ions results in distinct morphologies of mature 3-Dimensional vessel models.** 24 hours after either HUVEC or HBMEC were seeded into matrices they were exposed to 1Gy of each type of particle radiation and then cultured for a further 5 days until vessel structures had formed. Fixed cultures were stained for all protein material (DTAF – green) and nuclei (Propidium Iodide – red and imaging as yellow). Images are 10 slices 2 μm apart projected onto a single plane. **A**. Control HUVEC culture shows vessels with lumens that have formed a connecting network. **B**. HUVEC cultures exposed to 1 Gy Fe ions formed a network but vessels are often thinner without lumens (arrow). **C**. HUVEC cultures exposed to 1 Gy protons fail to form a network and vessels terminate in a dead end (arrow) Bar = 100 μm. **D**-**F**. HBMEC at a higher magnification. **D**. Control cultures form a network. **E**. cultures exposed to 1 Gy Fe ions formed a network but vessels are often thinner without lumens (arrow). **F**. Cultures exposed to 1 Gy protons fail to form a network and vessels terminate in a dead end (arrow). **G**. Quantitation based on length of vessel per cell shows a similar overall effect. Bar = 50 μm.

Low and high LET particle radiations inhibit vasculogenesis in human 3-D vessel models with the same relative biological effect [12]. Since high LET radiation is normally much more biologically effective in biological systems, this unusually high potency of low LET protons on vasculogenesis suggests that each type of radiation is stimulating distinct mechanisms of inhibition. We therefore examined in detail the morphologies of vessels treated with high LET Fe particles and low LET protons. Irradiations of 1Gy (energy = 1GeV) for each particle, were carried out 24 hours after plating of HUVEC and HBMEC in a 3-Dimensional matrix. Cultures were then assayed after maturation of vessel structures on day 6 (Figure 1). Control cultures developed capillary-like tubes with central lumens (Figure 1A and D). Cells exposed to Fe ions extended narrow cellular processes and made connections to other cells but did not develop a central lumen (Figure 1 B and E). Conversely, cells exposed to protons failed to make connections with other cells, cellular processes extended short distances into the gel matrix and terminated in a dead end (Figure 1 C and F). Both cell types responded to radiation in the same way. Overall inhibition of vessel formation was similar even though the morphologies were different. These morphologies suggest that protons inhibit motile tip extension during the formation of vascular guidance tunnels whereas Fe ions inhibit a later stage of vasculogenesis when endothelial cells migrate to form a tube - tubulogenesis.

### Inhibition of motile tip activity

**Figure 2:**
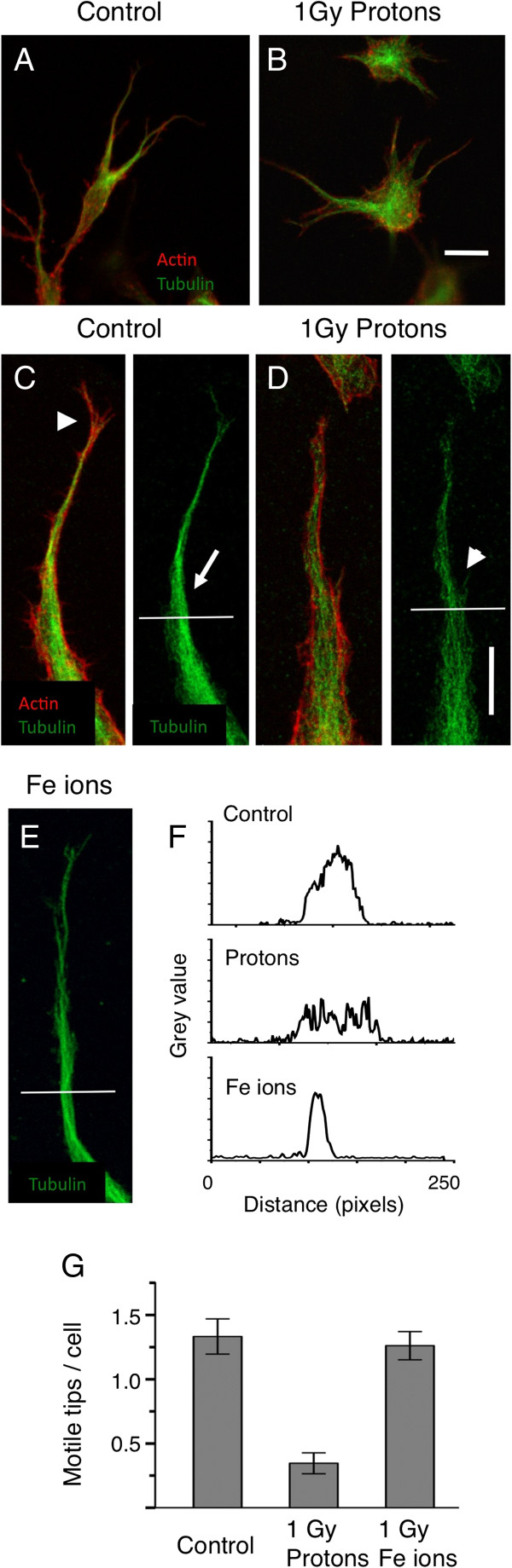
**High-energy protons but not high-energy Fe ions reduce the frequency of specialized motile tips of endothelial cells extending into the matrix. A** and **B** low magnification 3-D images (projected onto a single plane) of HBMEC 24 hours after plating and 2 hours after, sham exposure **A** and exposure to 1Gy of protons **B**. Cultures are stained for microtubules (green) and actin (red). Cells appear similar although microtubules are more concentrated in un-irradiated cultures. Bar = 25 μm. **C** and **D** Higher magnification in controls **C** reveals motile tips - streamlined cellular processes with motile actin structures such as filopodia (arrowhead) and bundled microtubules (arrow). In cultures exposed to 1 Gy of protons **D** cellular processes are less streamlined with unbundled microtubules (arrowhead). In cultures exposed to Fe ions **E** cellular processes contain bundled microtubules (arrow) as in the controls. Bar = 10 μm. **F**. Line scans measuring pixel brightness (grey value) along the horizontal lines shown in **C**-**E** demonstrate the determination of bundled microtubules (Materials and Methods), a single peak denotes bundled microtubules whereas multiple peaks seen after proton exposure reveals unbundled microtubules. **G**. Quantitation of processes with bundled microtubules shows that motile tips are inhibited 4-fold by protons but not by 1Gy of Fe ions.

We investigated the effects of these particles on the early stages of vasculogenesis by looking at the specialized motile tip of the extending HBMEC in 3-Dimensional matrices up to 24 hours after irradiation. As early as 2 hours after exposure we found differences in the actin and microtubule cytoskeletons (Figure 2). At this time the endothelial cells are extending cellular processes that are tipped by motile structures penetrating the matrix. Low power images do not show large differences between controls and those irradiated with 1 Gy high-energy protons (Figure 2 A and B) although the microtubule staining in the shaft of extending processes appeared to be more concentrated in the control cultures. High power microscopy however, revealed significant differences (Figures 2 C – F). The processes of the control cultures are streamlined and they contain tightly bundled microtubules that are associated with motile actin structures like filopodia. After treatment with 1 Gy protons the processes appeared less streamlined, actin filaments did not form filopodia and microtubules became unbundled. In contrast, cultures exposed to high-energy Fe ions were indistinguishable from controls. When the tips were quantitated (Materials and Methods) exposure to 1 Gy of high-energy protons but not 1 Gy of Fe ions resulted in a significant decrease in the number of these motile tips per cell (Figure 2 G). In contrast, exposure to high-energy Fe ions did not significantly reduce the frequency of motile tips (Figure 2 E). Thus these ionizing radiations induce different responses in the formation of motile structures. Protons inhibit motile structures while Fe ions do not.

### Matrix architecture

**Figure 3:**
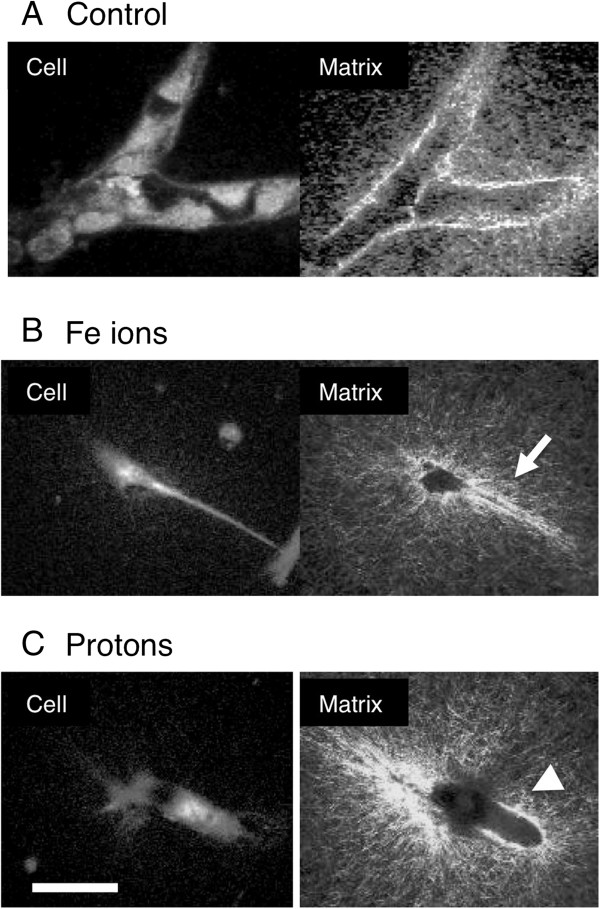
**Distinct modifications of the matrix architecture.** 24 hours after plating 3- Dimensional cultures of HBMEC were either unexposed **A** or exposed to 1Gy Fe ions **B** and 1 Gy protons **C**, then cultured for a further 5 days before fixation. Two-photon microscopy was used to visualize cell material stained with DTAF and collagen was visualized by Second Harmonic Generation microscopy (as shown). Control cultures have branched vessels with compressed bright staining collagen deposits surrounding them. Cultures exposed to Fe ions **B** display narrow guidance tunnels (arrow) surrounding narrow processes. Cultures exposed to protons **C** display shorter widened tunnels lined with compressed bright staining collagen deposits (Arrowhead). Bar = 50 μm.

Inhibition of motile tips at the early stages of vasculogenesis would be expected to result in a failure to form vascular guidance tunnels. On the other hand, inhibition of tubulogenesis would result in the earlier formation of guidance tunnels but not the later widening of these tunnels. We used SHG microscopy to visualize the collagen filaments in the matrix after HBMEC vessel maturation. Models were irradiated 24 hours after plating and then allowed to mature until day 6. In the control cultures branched vessels showed wide tunnels with bright staining collagen as a tube around the vessel (Figure 3 A). In cultures exposed to high LET Fe ions, we observed narrow guidance tunnels surrounding narrow processes (Figure 3 B) in addition to a number of narrow guidance tunnels without cellular material (not shown). Vessels had not matured into wide tunnels and then collapsed. In cultures exposed to low LET protons narrow guidance tunnels were not seen. Instead, shorter widened tunnels lined with bright staining collagen deposits were observed (Figure 3 C). Wide tunnels without cellular material were rare (not shown). Therefore the matrix architecture seen at the time of vessel maturation supports the notion that protons inhibit the early stages of motile tip cell activity and the formation of guidance tunnels while Fe ions inhibit the later stages of tubulogenesis.

### Rescue of vessel phenotype

**Figure 4:**
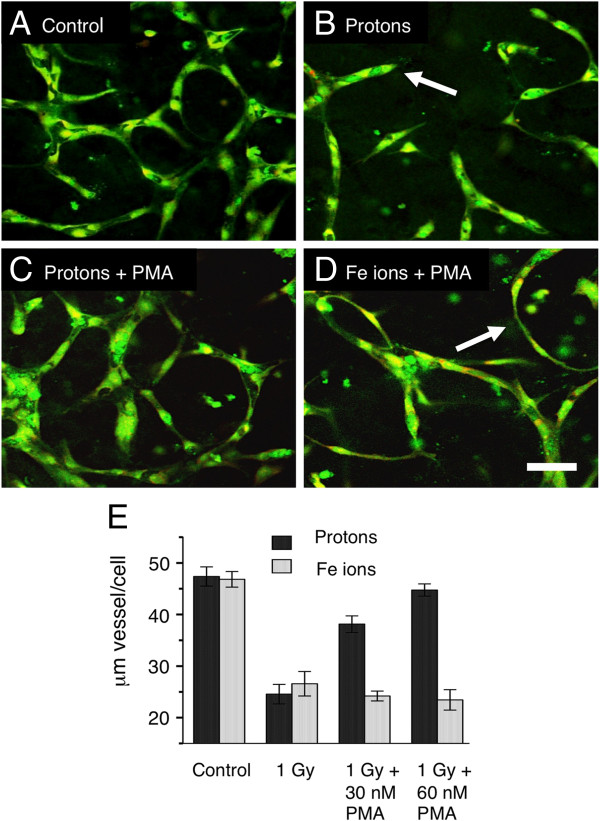
**Rescue of vessel formation at 5 days in endothelial cultures treated with high-energy protons and Fe ions.** HBMEC were allowed to begin vessel development for 24 hours. Cultures were then treated with PMA 15 minutes before irradiation. After maturation 5 days later, cultures were fixed and stained for all proteins (green) and for nuclei (red). **A**. Un-irradiated controls show robust vessel formation. **B**. 1 Gy Protons reduce vessel formation. Cellular processes extended short distances into the gel matrix and terminated in a dead end (arrow). **C**. 1 Gy protons and 30nM PMA, vessel growth is restored. **D**. Fe ions and 30nM PMA vessel growth is not restored. Cellular processes make connections but remain thin without lumens (arrow) similar to Fe ions without PMA (Figure 1). Bar = 50 μm. **E**. Quantitation of vessels under all conditions (length of vessel with lumen per cell). PMA rescues vessel formation inhibited by protons but not Fe ions.

Protein kinase C (PKC) has long been known to stimulate angiogenesis, it is in fact, added to the culture medium during our routine vessel culture (Materials and Methods). We hypothesized that either protons, Fe ions, or both may be inhibiting vasculogenesis in our model by inhibiting PKC since PKC isomers of different types are known to be second messengers involved in angiogenesis reviewed in [15]. Furthermore, rescue of the capillary phenotype might therefore be possible by stimulating PKC before irradiation. In our radiation experiments, PMA is included in the media at the initial plating and then 48 hours later (24 hours after exposure). To stimulate PKC efficiently we treated the cultures with an additional dose of PMA shortly before exposure. 30 nM and 60 nM phorbol ester (PMA) were added just 15 minutes before irradiation with protons and Fe ions. After irradiation, the developing vessels were cultured for a further 5 days as normal (with PMA) before fixation and assayed for capillary formation (Figure 4). Transient stimulation of PKC by PMA restored capillary formation in cultures irradiated with protons. Endothelial cells made connections and expanded to form vessel structures, which were similar to control cultures (Figure 4 A and C). In Fe ion irradiated cultures pretreated with PMA, the final morphology was indistinguishable from cultures irradiated without PMA (Figure 4 D and Figure 1 B and D), cells made connections but did not expand to form capillary like vessels. Quantitation of vessel growth showed that 30 nM and 60 nM PMA restored vessel growth around 75% and 100% respectively (Figure 4 D). Thus, the mechanisms for inhibition of vessel formation by protons and Fe ions must be distinct. These results indicate that inhibition of vessel formation by protons but not Fe ions at least in part involves the inhibition of PKC.

### Rescue of motile tip phenotype

**Figure 5:**
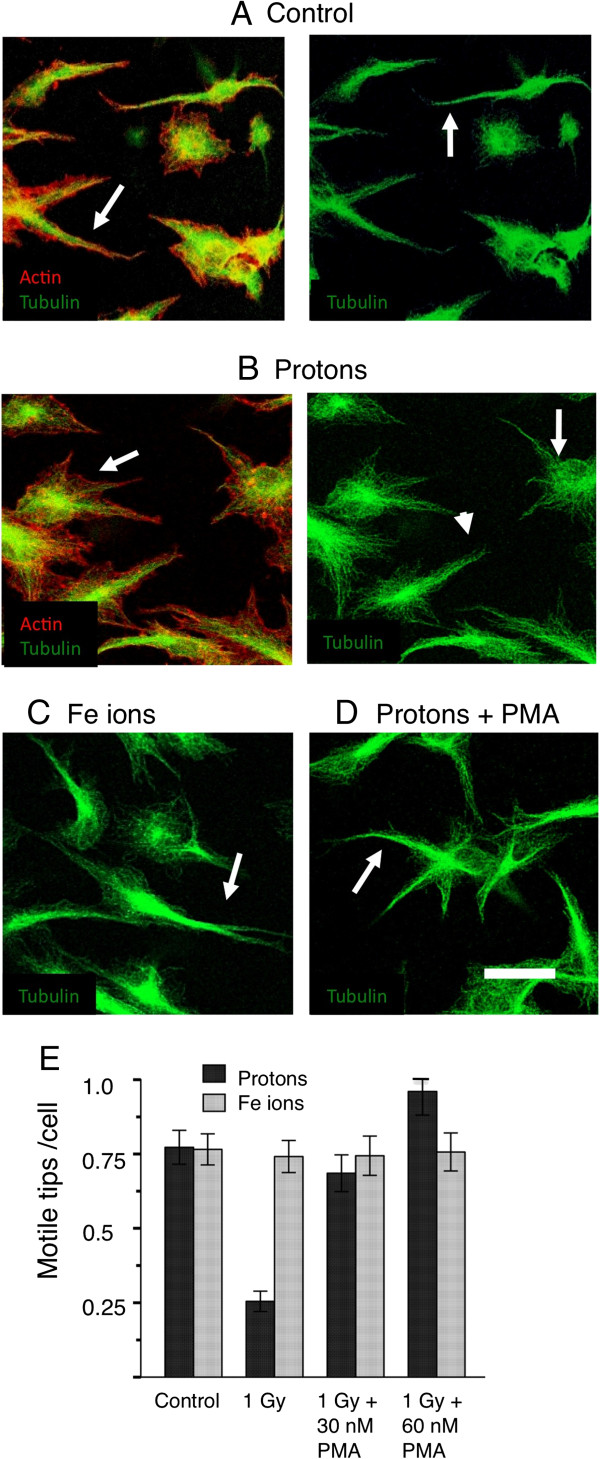
**Rescue of motile tips in endothelial cultures 2h post irradiation with high-energy protons and Fe ions.** HBMEC were allowed to begin vessel development for 24 hours. Cultures were then treated with PMA 15 minutes before irradiation. After 2 hours, cultures were fixed and stained for microtubules (green) and actin (red). In the extending cellular processes of control cultures **A**, F-actin was localized more closely to the microtubules and also present as filopodia (arrowhead). Microtubules were bundled (arrow). In the extending cellular processes of cultures irradiated with 1 Gy protons **B**, actin filaments showed fewer motile structures such as filopodia and a tendency to have spread away from the microtubules (arrowhead). microtubules were unbundled (arrow). Cultures treated with 1Gy Fe ions **C** resembled control cultures with bundled microtubules (arrow). **D** In the extending cellular processes of cultures treated with 1 Gy protons in the presence of 30 nM PMA, microtubules were bundled (arrow) PMA restored the motile tip morphology. Bar = 25 μm. **E**. Quantitation of motile tips under all conditions (Materials and Methods). PMA rescues motile tips formation inhibited by protons. Motile tips are not inhibited by Fe ions.

If PMA can restore vasculogenesis in proton-treated cultures and protons act by inhibiting the motility of tip cells, then the phorbol ester would also be expected to restore the cytoskeletal morphology of the motile tips. We tested this by treating HBMEC 3-Dimensional cultures with PMA for 15 minutes before irradiation and determining the number of motile tips 1.5 hours after irradiation, similar to the assay used in Figure 2. However, here we determined the number of motile tips by imaging those cells at the floor of the gel that were also partially resting on the substrate. Cytoskeletal morphology can be determined more readily using this approach since non-motile tips spread out more when in partial contact with the substrate while actively motile tips retain spear-shape with tightly bundled microtubules (Figure 5). This type of assay gave essentially the same results as the 3-Dimensional assay used in Figure 2.

We found that, as for vasculogenesis, stimulation of PKC by PMA was able to rescue the morphology of the motile tips in proton-treated cultures. Proton irradiation alone resulted in an absence of streamlined processes emerging from the cells. Microtubules were unbundled and actin filaments showed fewer motile structures such as filopodia and a tendency to have spread away from the microtubules (Figure 5B). Fe ion irradiation resulted in a morphology similar to the control with bundled microtubules (Figure 5C). Pretreatment with PMA restored the motile and streamlined phenotype of processes emerging from the cells (Figure 5D) since microtubules were bundled tightly similar to the control cultures seen in Figure 2. Quantitation of motile tips showed that PMA restored the number of motile tips per cell to levels seen in the controls and in Fe ion treated cultures. PMA did not however, elevate the number of motile tips in Fe ion cultures above that of the controls (Figure 5E).

## Discussion

We have shown here that low LET protons and high LET Fe ions inhibit the formation of model human brain capillaries by different mechanisms. In the case of protons, the inhibition involves the regulation of PKC-dependent motile tips leading to a failure of cellular processes to migrate through the matrix, form guidance tunnels, and meet up with other cell processes. In the case of Fe ions, inhibition does not involve the blockage of motile tip activity since these structures are not affected and cellular processes succeed in making guidance tunnels and connections. Instead, the cells fail to form widened tunnels in the matrix and lumen-containing tubular structures at the later stages of vasculogenesis.

An examination of the final vessel morphology, cytoskeletal arrangements, and matrix architecture, together with rescue of the motile tip phenotype by PMA, have efficiently distinguished between the inhibition of early and later stages of vasculogenesis by particles of different LET’s.

The notion that low LET protons inhibited the motility of tip cells penetrating the matrix is confirmed by the changes in the actin and microtubule skeletons, which play a major role in the formation of angiogenic sprouts reviewed in [16]. Unirradiated controls showed motile actin structures (filopodia) and bundled microtubules whereas proton-irradiated cells lost these features. The distal tip no longer had the characteristics ideally suited to cells that penetrate and grow through other tissues, that of spear-shaped and streamlined protrusions containing bundled microtubules. To our knowledge, this is the first report of bundled microtubules in motile tip cells although they are remarkably similar to the bundled microtubules in other cells that grow through tissue, such as rapidly growing axonal growth cones [17] and cancer cells making an epithelial/mesenchyme transition [18]. Cells exposed to Fe ions displayed motile tip features and were able to form a network although later stages were inhibited.

Inhibition of tip cell activity is confirmed by visualization of the collagen matrix. A fundamental mechanism in vessel tube formation is the matrix type 1-metalloproteinase dependent creation of a network of guidance tunnels, which serve as conduits for later events of endothelial cell migration and tube remodeling [19]. SHG microscopy has been used to show that only the wider mature vessels have increases in collagen density around the perimeter of tunnels [20]. The matrix protein in these areas is anisotropically altered suggesting that collagen was displaced or compacted during tube and lumen formation. Our observations with SHG show that high LET Fe ions halt vessel development after guidance tunnel formation but before tubulogenesis. Narrow guidance tunnels with or without cell processes were evident while wider collagen lined tunnels were absent. Low LET protons inhibit vessel formation during guidance tunnel stage but then tubulogenesis continues even though there is a much reduced guidance tunnel network. Narrow guidance tunnels were absent while wider collagen lined tunnels were present.

The difference between the two types of inhibition was further confirmed by the selective rescue of vessel phenotype, and of tip motility by the use of PMA to stimulate PKC immediately before irradiation. Protein Kinase C isomers of different types are known to be second messengers involved in angiogenesis (reviewed in 15) and studies have implicated Protein Kinase C in vessel formation and the effects of radiation [21,22]. However, further studies are required to show that inhibition of PKC is the cause of the effect of protons. Although the use of PMA here does not give any new information on the role of specific PKC’s in vasculogenesis, it has proved useful for distinguishing between inhibition of the early stages by low LET ions and the later stages by high LET ions. It also reveals that the effect is transient. Development of vessels is resumed after further culture (including PMA treatment) since wider tubes with lumens are eventually formed even though the extent of the network has been limited.

Although most studies on radiation and the vasculature have been carried out using sources that produce low LET electrons (gamma photons and X-rays), a comparison with studies on low LET particles reviewed in [23] show that several biological responses of protons including angiogenesis, are different or even opposite. Our own observations show that protons are at least 8 times more effective than gamma rays at inhibiting vasculogenesis [12]. Furthermore, protons have been shown to down-regulate the expression of pro-angiogenic factors like VEGF, in addition to invasion, in endothelial cells and fibroblasts [13]. This is one possible mechanism whereby protons could be inhibiting vasculogenesis in the present study. However, the mechanistic basis for the difference in low LET proton response versus low LET electron response remains a puzzle.

The effect of high LET Fe ions was more insidious and longer lasting. The initial motility during the first 24 hours after irradiation appeared to be unaffected while later development was inhibited. Therefore, the adverse effects of these heavy ions lasted much longer than those of the low LET protons. Although there are relatively few studies on the effects of high LET ion particles on vessel formation, they support the notion that these particles inhibit vessel formation. The effect of 290 MeV carbon ions (LET 110 KeV/μm) was examined on developing vessel models [9] indicating sensitivity to heavy ions, with a low dose (10 cGy) of carbon ions inhibiting vessel formation in addition to cell migration. In vivo, the effects of Fe particles on mouse hippocampal microvessels was examined [11], and it was found that a dose as low as 50 cGy resulted in loss of endothelial cells 1 year after irradiation. The mechanism for heavy ion inhibition is also not well known. A tip cell with average morphology exposed to a dose of 75 cGy is estimated to get approximately 42 Fe ion particle traversals compared to 25000 traversals by high LET protons. For protons, the energy deposition is spread over the cell more evenly and this may facilitate global effects on cellular signaling. For Fe ions, a few sites receive much more concentrated, and therefore locally damaging, energy depositions. Apoptosis is unlikely since we have previously shown that doses of Fe ions greater than 1 Gy and doses of protons greater than 2 Gy are necessary to induce apoptosis as detected by Terminal deoxynucleotidyl transferase dUTP nick end labeling (TUNEL) assay [12]. One possible mechanism is the downstream effects of DNA damage. Heavy ions are known to cause complex DNA damage [24], which is persistent in these HUVEC 3-D cultures [25]. Other possibilities are, mitochondrial damage and apoptosis signaling that occurs prior to DNA fragmentation.

In the space environment, humans will be exposed to a variety of ion particles with a range of LET’s.. Recently, measurements of energetic particle radiation were made on the Mars Science Laboratory spacecraft, containing the Curiosity rover. For a short round trip (360 days) the total dose of heavy particles was found to be 17.2 cGy and a dose equivalent of 662 mSieverts, with additional variable contributions from solar particle events [26]. These daily doses are lower than those that will inhibit vasculogenesis, although the existence of reduced vasculature in mice one year after exposure [11], suggests that damage might be accumulative.

Solar particle event (SPE) dose-rates, can vary between 0 and 100 mGy/h in a spacecraft or up to 500 mGy/h for an astronaut exposed outside the vehicle in deep space or on the Moon’s surface [27]. In this case, doses high enough to inhibit vasculogenesis could be achieved even in deeper tissues like bone marrow [4]. SPE’s contain protons of mixed energy and therefore mixed LET’s, some, could be low enough to inhibit the early stages of angiogenesis and others high enough to inhibit the later stages. We are currently investigating the LET ranges for each type of inhibition to determine the contribution of each type of radiation in the space environment. The existence of distinct mechanisms of the inhibition of vasculogenesis according to LET, raises the possibility that normal angiogenic repair could be inhibited by two different species of particles, and that these effects could be additive or even synergistic. Furthermore, if the radiation also damages the endothelial barrier, thereby creating the need for more angiogenic repair, the harmful effects of space radiation to the vasculature could be further compounded.

For particle radiotherapy, the inhibition of tumor vasculature would be desirable. The results shown here raise the possibility that mixed particle therapies with defined LET ranges might target different stages of angiogenesis and therefore be more effective at inhibiting tumor vessel growth. Also, a combination of specific anti-angiogenic drugs and particle radiation of specific LETs could efficiently target selected stages of angogenesis.

## Conclusions

We have used assays that have enabled us to distinguish between the inhibition of an early stage and a late stage of vasculogenesis. The unexpected high relative biological effectiveness of low LET protons compared to high LET Fe ions on vessel formation, can be explained by the fact that particle radiation can inhibit vasculogenesis in two distinct ways according to the LET of the radiation. Low LET protons, which deposit energy more evenly throughout the cell, inhibit the early stages during the extension of motile tips and the formation of guidance tunnels. High LET Fe ions do not inhibit the early stages only the later stages of tube formation. These results have implications for both human space travel and for particle radiotherapy.

## Competing interests

The authors declare that they have no competing interests.

## Authors’ contributions

PG Carried out; conception and design of the work, experimentation at Brookhaven National Laboratory, acquisition of data, and analysis and interpretation of data. PS Carried out; acquisition of data, experimentation at Brookhaven National Laboratory, and analysis and interpretation of data. AB Carried out acquisition of data using the Two-photon and second harmonic generation (SHG) microscopy. CG Carried out conception and analysis and interpretation of data. All authors read and approved the final manuscript.
